# The Impact of Demographic, Psychosocial, and Socioeconomic Factors on Barriers to Accessing Dental Services in Bulgaria

**DOI:** 10.7759/cureus.67011

**Published:** 2024-08-16

**Authors:** Stanislav M Nenov, Boyko K Bonev

**Affiliations:** 1 Dental Public Health, Faculty of Dental Medicine, Medical University - Sofia, Sofia, BGR

**Keywords:** self-esteem, socioeconomic factors, psychosocial factors, dental status, dental services, dental health, demography, barriers

## Abstract

Introduction

Dental health is an important component of overall health. Many factors can obstruct access to dental care and limit the utilization of services. Barriers to accessing dental services are divided into three groups - by patients, by dental profession, and by state and society. Factors by patients are proven to be the leading ones.

Methods

We conducted an anonymous survey among 416 Bulgarians to study the barriers to accessing dental services and the demographic, psychosocial, and socioeconomic factors that influence those barriers. The research complies with ethical standards and is approved by the Ethics Committee of Medical University, Sofia.

Results

The main group of barriers to accessing dental care in the Republic of Bulgaria was patient-related (67.03%). They led all groups by gender, age, residence, education, income, overall health status, self-assessment of dental health, and frequency of visits. Barriers by state and society were second in importance (28.9%) and were mentioned mainly among men, low-income people (33.96%), the less educated (27.33%), age group 45-65 years (22.76%), and patients visiting a dental office only in case of emergency (32.97%). The leading reason for the postponement of visits was lack of pain (31.21%). Lack of pain was more often indicated among women (20.04%), age group 45-65 years (28.28%), and rural population (31.04%). The cost of dental treatment (15.54%) was not a significant factor and was outweighed by psychosocial factors such as lack of time (17.8%) and dental fear and anxiety (16.67%). Dental fear and anxiety were cited mainly among women (9.12%), younger patients (17.9%), the less educated (12.21%), those with low income (9.62%), and those without income (25%), as well as among people with low self-estimation of their oral health status (40%) and those visiting a dental office irregularly (25.53%).

Conclusion

The main group of barriers to accessing dental services in the Republic of Bulgaria was those created by patients and were indicated mainly among women, people with higher education and income, and those from rural populations, while barriers by state and society were indicated mainly by men, low income, less educated, and people over 45 years. Complex impact by more than one group of factors was reported mainly by middle-aged people, city populations, people visiting a dental office irregularly, and those with low self-assessment of their dental status. Patients postponed dental treatment mainly due to lack of pain, which was more significant among women, people over 45 years, and rural populations. The cost of dental treatment is no longer a significant factor and has been overtaken by psychosocial factors such as lack of time and dental fear and anxiety.

## Introduction

Dental health is a key indicator of overall health, quality of life, and well-being [1]. Access to dental care is defined as “the use of services in accordance with real health needs” [2]. It could be studied in terms of accessibility, availability, acceptability, and adequacy of the provided dental activities [3,4]. Barriers to access are factors that hinder the utilization of dental services [5]. They can be classified as predisposing factors, enabling factors, and needs. Each group consists of modifiable and non-modifiable factors [6]. Barriers to access impact the motivation of patients to visit dental offices [7]. Lack of understanding of the negative effect of the obstructing factors could limit the improvement of access to services [8].

According to the World Dental Federation (FDI), barriers are classified into three main categories - by patients, by dental profession, and by state and society [9]. This classification corresponds to the psychosocial nature of factors and facilitates their understanding by both patients and dentists [10]. Four kinds of barriers are experienced by patients - dental fear and anxiety, cost of treatment, unperceived treatment needs, and lack of access to services [9]. The link between negative experiences with dental procedures and wrong perceptions and attitudes about treatment has been proven and is perceived by patients as a barrier to future treatment [11].

Barriers by dental profession include uneven territorial distribution of practitioners, number of dentists who are non-responsive to the needs of the population of a particular region, education and training that does not meet the changing needs of the population, and insufficient sensitivity to attitudes and needs of patients [9].

Obstructing factors by state and society are insufficient public encouragement of healthy oral behavioral practices, irrational organization of the dental healthcare system, inadequate planning and training of dental personnel, lack of health education and poor health knowledge of the population, and insufficient support for dental scientific research [10].

## Materials and methods

The purpose of this study was to systematically identify and analyze barriers to accessing dental services in the Republic of Bulgaria, focusing on demographic, psychosocial, and socioeconomic factors that influence access to dental care. To achieve that purpose, we conducted a cross-sectional study by using an anonymous self-administered questionnaire. The study design and questionnaire were approved by the Ethics Committee of Medical University, Sofia (approval no. 02/24.02.2019), and the survey was conducted in accordance with the World Medical Association Declaration of Helsinki as revised in 2013.

We performed a pilot study in September 2020 among 50 patients from Sofia to determine the clarity, content, and face validity of the survey. Subsequently, final questionnaires were administered to the whole sample (n=416, response rate=100%), between November 2020 and March 2021.

We conducted the study in dental offices located in six major municipalities corresponding to economic regions of the country and collected the primary data during dental checkups. We applied a systematic random sampling technique to recruit study participants. All patients who gave voluntary consent to take part in the study were included in the sample. Moreover, being representatives of the employed part of the population (aged 18-65 years) was an additional inclusion criterion. Being aged under 18 or over 65 years was the criteria for exclusion. Prior to completing the questionnaire, patients were briefly explained about the study's purpose, methods, expected results, and possible implications for research and practice. They were additionally informed that participation was anonymous and voluntary, as well as that confidentiality of data was guaranteed. All patients signed informed consent and then were asked to complete the questionnaire.

Half of the participants lived in Sofia, the capital of the Republic of Bulgaria - 209 (50.24%). The remainder lived in the following municipalities: Vratsa - 70 (16.83%), Veliko Tarnovo - 48 (11.54%), Plovdiv - 40 (9.62%), Burgas - 29 (6.97%), and Yambol - 20 (4.81%). The proportion of participants corresponded to the proportion of the population living in different economic regions of the country. Most participants - 383 (92.03%) lived in cities, and only 29 (6.97%) lived in rural areas. Participants were predominantly women - 230 (55.29%), while men totaled 186 (44.71%). For the purposes of the study, participants were divided into three age groups: 18-29 years (96, 23.08%); 30-44 years (175, 42.07%); and 45-65 years (145, 35.86%).

The main instrument of the current study consisted of 19 questions divided into two sections. The first (passport) part included 11 questions to determine patients’ sociodemographic status - age (Q1), gender (Q2), place of residence (Q3), level of education (Q4), amount of income (Q5), self-assessment of health status regarding comorbidities (Q6), self-assessed level of general (Q7) and dental (Q8) health status, and finally, attendance patterns-last visit (Q9), reasons (Q10), and regularity (Q11).

The second part consisted of eight multiple-choice questions regarding barriers to accessing dental services from different categories (main barriers and reasons) (Q12) and especially the barriers specific to patients themselves (Q13) - fear and anxiety (Q14), lack of pain (Q15), cost of treatment (Q16), lack of access (Q17), negative experience (Q18), and lack of time (Q19). These variables were selected for the current investigation as they have been suggested multiple times in the literature as factors limiting access to dental services [9-11].

We grouped and analyzed the primary data with statistical software programs R (The R Foundation for Statistical Computing, Vienna, Austria) and IBM SPSS Statistics v.27.0 (IBM Corp., Armonk, USA). We used standard descriptive statistics to calculate values and distribution of variables, as well as distribution of the main groups of barriers, frequency of different barriers specific to patients themselves, and reasons for the occurrence of each barrier. Furthermore, we compared distributions of barriers to accessing dental services in different groups according to demographic, socioeconomic, and psychosocial characteristics. To prove significant associations of barriers to accessing dental services with examined variables (age, gender, residence, education, income, health status, self-assessment of dental health, and frequency of visits), Pearson’s chi-square test of homogeneity (χ2) was applied. Finally, to prove the power and direction of associations among different variables, Pearson’s r (correlation analysis) was used. The significance level was set at p-value <0.05.

## Results

**Figure 1:**
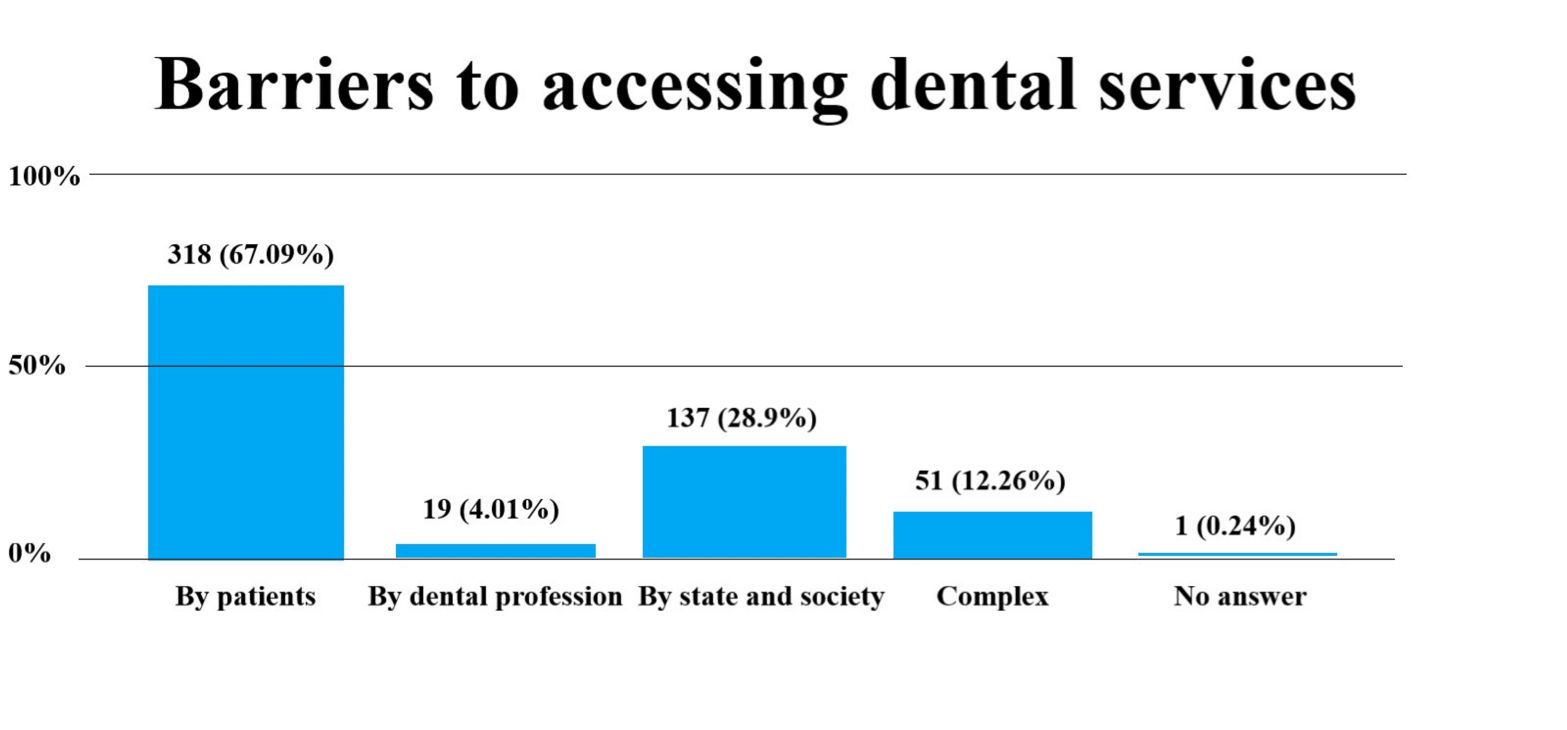
Barriers to accessing dental services

We found that patient barriers were the main category of barriers to accessing dental services: 318 (67.09%). That was followed by barriers imposed by state and society: 137 (28.9%). Barriers by dental profession had the lowest impact rate: 19 (4.01%). A complex effect of more than one category was reported by 51 participants (12.26%), while one participant (0.26%) did not respond to the question (Figure 1).

Barriers by patients led in both genders as well as in all groups by age, residence, education, and income. Barriers related to state and society were mainly indicated in the low-income group (less than 610) - 18 (33.96%), age group 45-65 years - 33 (22.76%), and the group without higher education - 47 (27.33%). Complex influence on all groups of obstructing factors was found mainly among men - 25 (13.44%), age group 30-44 years - 26 (14.94%), and urban population - 47 (12.3%). Our analysis of variance proved the link between the level of education and barriers to accessing dental services (Pearson’s chi-square, p-value < 0.05). Barriers by state and society were claimed mainly by individuals with lower education - 47 (27.33%), while multiple barriers were indicated by twice as many participants with higher education - 38 (15.64%) than by those without - 13 (7.56%) (Table 1).

**Table 1:** Dependence of the barriers to accessing dental services on the demographic characteristics of populations Income is in Bulgarian currency

	Main group of barriers to accessing dental services / Number (% members of group)				χ^2^ (Pearson’s chi-square test of homogeneity)		
	By state and society	By dental profession	By patients	Complex	χ^2^	df	p-value
Gender							
Male	44 (23.66%)	1 (0.54%)	116 (62.37%)	25 (13.44%)	2	3	0.5
Female	47 (20.52%)	4 (1.75%)	152 (66.38%)	26 (11.35%)			
Overall	91 (21.93%)	5 (1.21%)	286 (64.58%)	51 (12.29%)			
Age							
18-29	20 (20.83%)	2 (2.08%)	62 (64.58%)	12 (12.5%)	4	6	0.7
30-44	38 (21.84%)	1 (0.57%)	109 (62.64%)	26 (14.94%)			
45-65	33 (22.76%)	2 (1.38%)	97 (66.9%)	13 (8.97%)			
Overall	91 (21.93%)	5 (1.21%)	268 (68.92%)	51 (12.29%)			
Residence							
Urban area	84 (21.99%)	5 (1.31%)	246 (64.4%)	47 (12.3%)	0.6	3	0.9
Rural area	6 (20.69%)	0 (0%)	20 (68.97%)	3 (10.35%)			
Overall	90 (21.9%)	5 (1.22%)	266 (64.72%)	50 (12.17%)			
Education							
Higher	44 (18.11%)	3 (1.24%)	158 (65.02%)	38 (15.64%)	9	3	0.03
Lower level	47 (27.33%)	2 (1.16%)	110 (63.95%)	13 (7.56%)			
Overall	91 (21.9%)	5 (1.21%)	268 (64.58%)	51 (12.29%)			
Income							
Without	4 (14.29%)	0 (0%)	20 (71.43%)	4 (14.29%)	27	21	0.2
< 610	18 (33.96%)	0 (0%)	29 (54.72%)	6 (11.32%)			
610-1000	17 (23.94%)	0 (0%)	50 (70.42%)	4 (5.63%)			
1000-1500	24 (25.53%)	3 (3.19%)	54 (57.45%)	13 (13.83%)			
1500-2000	7 (11.11%)	0 (0%)	46 (73.02%)	10 (15.87%)			
2000-2500	10 (27.03%)	0 (0%)	21 (56.76%)	6 (16.22%)			
2500-3000	4 (16.67%)	0 (0%)	18 (75%)	2 (8.33%)			
> 3000	5 (13.89%)	1 (2.78%)	25 (69.44%)	5 (13.89%)			
Overall	89 (21.92%)	4 (0.96%)	263 (64.78%)	50 (12.32%)			

We explored the link between the subjective assessment of dental health by participants and barriers to accessing dental services. Our analysis showed that barriers by patients were the leading ones. They were reported by three times more people with good overall health - 200 (66.45%) than by those with comorbidities - 68 (60.18%). The same group of barriers was most frequently identified by persons assessing their dental health as “excellent” - 79 (75.96%), but was significantly less indicated by patients visiting dental offices only in case of emergency - 49 (53.85%). Barriers by state and society were more often indicated by patients with comorbidities - 26 (23.01%), by those assessing dental health as “satisfactory” - 22 (34.92%), and by people visiting the dentist only when they felt pain - 30 (32.97%).

Our statistical analysis showed a significant difference in the distribution of barriers to accessing dental services in relation to self-estimation of dental health and frequency of visits to a dental office (Pearson’s chi-square, p-value < 0.05). The significance of the link between variables is proven by correlation analysis (Pearson’s r; p < 0.05) and shows that barriers by state and society were mainly associated with people with poor self-assessment of dental status and those who rarely visited the dentist, while barriers by patients were mainly indicated by the those frequently visiting the dentist (Table 2).

**Table 2:** The link between barriers to accessing dental services and subjective factors

	Main group of barriers to accessing dental services / Number (% members of group)				χ^2^ (Pearson’s chi-square test of homogeneity)		
	By state and society	By dental profession	By patients	Complex	χ^2^	df	p-value
Health status							
With comorbidities	26 (23.01%)	1 (0.89%)	68 (60.18%)	18 (15.93%)	2	3	0.5
Without comorbidities	65 (21.6%)	3 (1%)	200 (66.45%)	33 (10.96%)			
Overall	91 (21.98%)	4 (0.97%)	268 (64.73%)	51 (12.32%)			
Self-assessment of dental health							
Excellent	16 (15.39%)	0 (0%)	79 (75.96%)	9 (8.65%)	26	9	0.002
Good	53 (21.81%)	3 (1.24%)	155 (63.79%)	32 (13.17%)			
Satisfactory	22 (34.92%)	2 (3.18%)	32 (50.79%)	7 (11.11%)			
Bad	0 (0%)	0 (0%)	2 (40%)	3 (60%)			
Overall	91 (21.93%)	5 (1.21%)	268 (64.58%)	51 (12.29%)			
Pearson Correlation	-0.12	0.04	0.05				
p	0.02	0.46	0.37				
Frequency of dental visits							
6 months	14 (14.58%)	0 (0%)	71 (73.96%)	11 (11.46%)	18	9	0.04
Annually	40 (22.1%)	1 (0.55%)	116 (64.09%)	24 (13.26%)			
Several years	7 (14.89%)	2 (4.26%)	32 (68.09%)	6 (12.77%)			
Emergency	30 (32.97%)	2 (2.2%)	49 (53.85%)	10 (10.99%)			
Overall	91 (21.93%)	5 (1.21%)	268 (64.58%)	51 (12.29%)			
Pearson Correlation	-0.13	0.04	0.15				
p	0.006	0.48	0.003				

**Figure 2:**
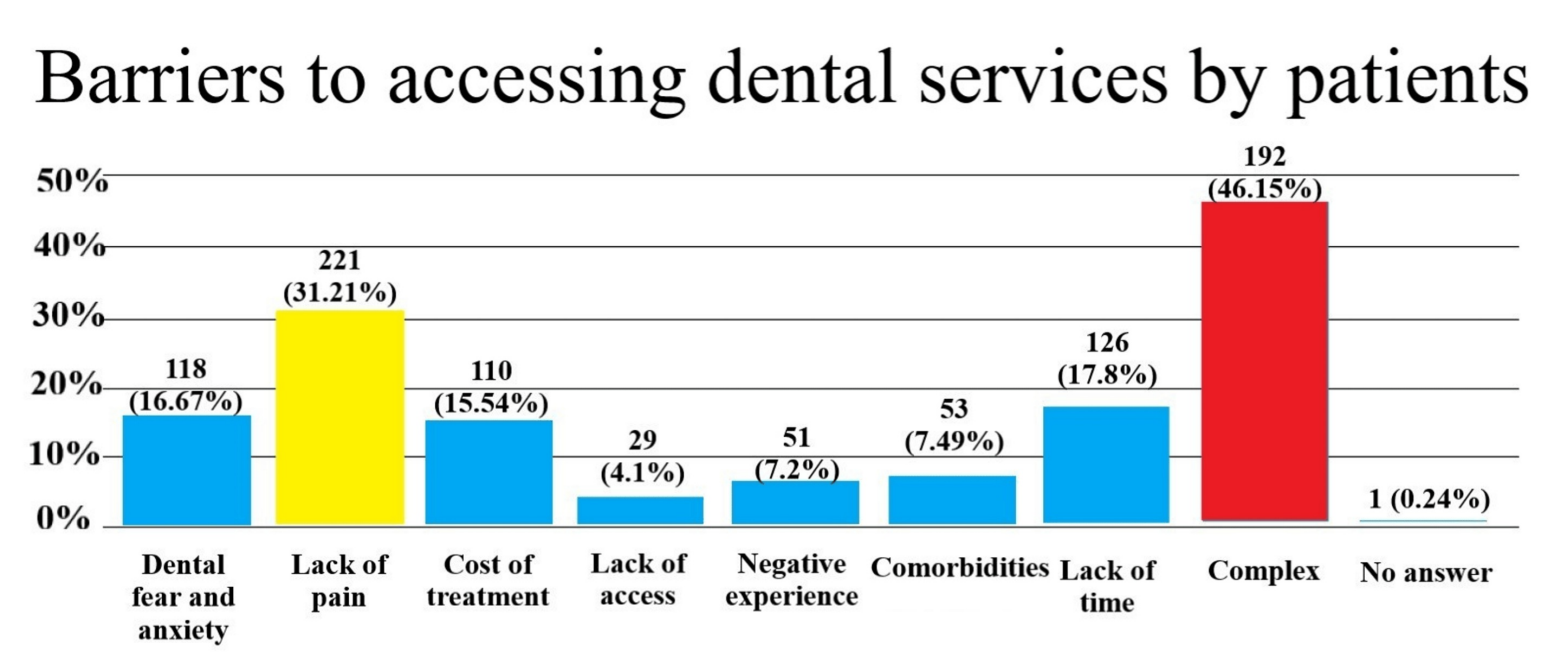
Barriers to accessing dental services by patients

The main reason for patients’ postponing dental treatment was lack of pain - 221 (31.21%), followed by lack of time - 126 (17.8%). The factor with the lowest impact rate was lack of access to services - 29 (4.1%). More than one reason was stated by 192 participants (46.15%), while one (0.26%) did not answer the question (Figure 2).

The main reason for experiencing dental fear was pain during manipulation - 171 (30.54%), followed by unpleasant thoughts and feelings about the treatment - 76 (13.57%), unreasonable fear - 67 (11.96%), and fear of the specific manipulation - 58 (10.36%). The complex impact of barriers was indicated by 105 (25.25%) of participants. The leading reason for not visiting a dental office was lack of pain - 269 (50.28%). Other important factors were negligence in seeking dental prevention - 152 (28.41%) and lack of knowledge about dental health and diseases - 58 (10.84%). More than one factor was cited by a quarter of participants - 104. The cost of treatment was perceived as a barrier mainly due to the high price of dental treatments - 166 (37.05%) and to complementary subjective factors (e.g., low income and other priorities) - 96 (21.43%). More than one reason was mentioned by 64 patients (15.39%). The main factors obstructing access to services were remoteness of dental practice from home or workplace - 110 (29.73%) as well as difficulties in making an appointment - 82 (22.16%). The negative experience of patients was mainly due to previous unsuccessful treatment - 172 (41.15%) and inappropriate behavior of the dentist - 89 (21.29%).

Complex impact by more than one barrier was reported by 55 patients (13.22%). The complex influence of multiple factors related to the patients led all groups by gender, age, education, income, and urban residence. The main barrier for the rural population was lack of dental pain - 9 (31.04%). It was the second most important reason for the postponement of treatment in the groups according to gender and age. This barrier was more often noted by women - 55 (24.02%) than by men - 36 (19.36%) and in the age group 45-65 years - 41 (28.28%). Dental fear and anxiety were reported two times less in the group with higher education - 15 (6.17%) than in the group without - 21 (12.21%), and they were most frequent among patients without income - 7 (25%) and with low income (610-1000) - 10 (14.09%). Analysis of variance revealed a significant difference in the distribution of barriers to access for patients according to age and income (Pearson’s chi-square, p-value < 0.05) (Table 3).

**Table 3:** Main barriers to accessing dental services by patients in relation to sociodemographic characteristics Income is in Bulgarian currency

	Main barrier to accessing dental services by patients / Number (% members of group)								χ^2^ (Pearson’s chi-square test of homogeneity)			
	Complex	Cost of treatment	Dental fear and anxiety	Lack of access	Lack of pain	Lack of time	Co-morbidities	Bad experience	χ^2^	df	p-value	
Gender												
Male	92 (49.46%)	7 (3.76%)	15 (8.05%)	3 (1.61%)	36 (19.36%)	27 (14.52%)	6 (3.23%)	0 (0%)	10	7	0.2	
Female	100 (43.67%)	19 (8.41%)	21 (9.17%)	3 (1.31%)	55 (24.02%)	22 (9.61%)	6 (2.62%)	3 (100%)				
Overall	192 (46.27%)	26 (6.27%)	36 (8.68%)	6 (1.45%)	91 (21.93%)	49 (11.81%)	12 (2.89%)	3 (0.72%)				
Age												
18-29	42 (44.21%)	3 (3.16%)	17 (17.9%)	0 (0%)	16 (16.84%)	11 (11.58%)	5 (5.26%)	1 (1.1%)	43	14	9е^-05^	
30-44	95 (54.29%)	13 (7.43%)	4 (2.29%)	6 (3.43%)	34 (19.43%)	19 (10.86%)	2 (1.14%)	2 (1.14%)				
45-65	55 (37.29%)	10 (6.9%)	15 (10.35%)	0 (0%)	41 (28.28%)	19 (13.1%)	5 (3.45%)	0 (0%)				
Overall	192 (46.27%)	26 (6.27%)	36 (8.68%)	6 (1.45%)	91 (21.93%)	49 (11.81%)	12 (2.89%)	3 (0.72%)				
Residence												
Urban area	180 (47.12%)	23 (7.59%)	31 (8.12%)	6 (1.57%)	80 (20.94%)	47 (19.37%)	12 (3.14%)	3 (0.79%)	7	7	0.5	
Rural area	12 (20.69%)	1 (3.45%)	5 (17.24%)	0 (0%)	9 (31.04%)	2 (6.9%)	0 (0%)	0 (0%)				
Overall	192 (46.27%)	24 (5.84%)	36 (8.76%)	6 (1.46%)	89 (21.65%)	49 (11.92%)	12 (2.92%)	3 0.73%				
Education												
Higher	124 (51.03%)	16 (6.58%)	15 (6.17%)	4 (1.65%)	49 (20.17%)	27 (11.11%)	6 (2.47%)	2 (0.82%)	9	7	0.3	
Lower	68 (39.54%)	10 (5.81%)	21 (12.21%)	2 (1.16%)	42 (24.42%)	22 (12.79%)	6 (3.49%)	1 (0.58%)				
Overall	192 (46.27%)	26 (6.27%)	36 (8.68%)	6 (1.45%)	91 (21.93%)	49 (11.81%)	12 (2.89%)	3 (0.72%)				
Income												
Without	7 (25%)	3 (10.71%)	7 (25%)	1 (3.57%)	5 (17.86%)	2 (7.14%)	3 (10.17%)	0 (0%)	78	49	0.005	
< 610	28 (53.85%)	1 (1.92%)	5 (9.62%)	0 (0%)	11 (21.15%)	7 (13.46%)	0 (0%)	0 (0%)				
610-1000	26 (36.62%)	7 (9.86%)	10 (14.09%)	1 (1.41%)	17 (23.94%)	8 (11.27%)	2 (2.82%)	0 (0%)				
1000-1500	42 (44.68%)	11 (11.7%)	8 (8.51%)	1 (1.06%)	24 (25.53%)	4 (4.26%)	2 (2.13%)	2 (2.13%)				
1500-2000	32 (50.79%)	1 (1.59%)	4 (6.35%)	0 (0%)	16 (25.4%)	8 (12.7%)	2 (3.18%)	0 (0%)				
2000-2500	21 (56.76%)	1 (2.7%)	1 (2.7%)	0 (0%)	3 (8.11%)	9 (24.32%)	1 (2.7%)	1 (1.59%)				
2500-3000	16 (52%)	1 (4%)	0 (0%)	1 (4%)	7 (28%)	2 (8%)	1 (4%)	0 (0%)				
> 3000	19 (52.78%)	0 (0%)	1 (2.78%)	2 (5.56%)	7 (19.44%)	7 (19.44%)	0 (0%)	0 (0%)				
Overall	188 (46.31%)	25 (6.16%)	36 (8.87%)	6 (1.48%)	90 (22.17%)	47 (11.58%)	11 (2.71%)	3 (0.74%)				

The complex impact of multiple factors led all groups by health status, self-assessment of dental status, and frequency of visits. Lack of pain was the second most important reason for postponement of treatment for patients with comorbidities - 31 (27.43%) as well as for healthy patients - 60 (19.93%). Lack of pain was the second most important reason also in all groups by frequency of visits and self-assessment of dental status except the group with bad evaluation of dental health in which dental fear is the second of importance - 2 (40%). Dental fear was more frequent among patients with comorbidities - 31 (10.3%) and among those visiting a dental office irregularly - 12 (25.53%). Analysis of variance proved the significance of the link between the frequency of dental visits and barriers to access to services (Pearson’s chi-square, p-value < 0.05) (Table 4).

**Table 4:** Main barriers to accessing dental services in relation to subjective factors

	Main barrier to accessing dental services by patients / Number (% members of group)								χ^2^ (Pearson’s chi-square test of homogeneity)			
	Complex	Cost of treatment	Dental fear and anxiety	Lack of access	Lack of pain	Lack of time	Co-morbidities	Bad experience	χ^2^	df	p-value	
Health status												
With comorbidities	54 (47.79%)	9 (7.97%)	5 (4.42%)	2 (1.77%)	31 (27.43%)	8 (7.08%)	4 (3.54%)	0 (0%)	10	7	0.2	
Without comorbidities	138 (45.85%)	17 (6.65%)	31 (10.3%)	4 (1.33%)	60 (19.93%)	40 (13.29%)	8 (2.66%)	3 (1%)				
Overall	192 (46.38%)	26 (6.28%)	36 (8.7%)	6 (1.45%)	91 (21.98%)	48 (11.59%)	12 (2.9%)	3 (0.73%)				
Self-assessment of dental health												
Excellent	39 (37.86%)	5 (4.85%)	13 (12.62%)	1 (0.97%)	25 (24.27%)	15 (14.56%)	5 (4.85%)	0 (0%)	21	21	0.4	
Good	118 (48.36%)	15 (6.15%)	19 (7.79%)	4 (1.64%)	54 (22.13%)	26 (10.66%)	5 (2.05%)	3 (1.23%)				
Satisfactory	33 (52.38%)	6 (9.52%)	2 (3.18%)	1 (1.59%)	12 (19.05%)	7 (11.11%)	2 (3.18%)	0 (0%)				
Bad	2 (40%)	0 (0%)	2 (40%)	0 (0%)	0 (0%)	1 (20%)	0 (0%)	0 (0%)				
Overall	192 (46.27%)	26 (6.27%)	36 (8.68%)	6 (1.45%)	91 (21.93%)	49 (11.81%)	12 (2.89%)	3 (0.72%)				
Frequency of dental visits												
6 months	29 (29.9%)	9 (9.28%)	12 (12.37%)	2 (2.06%)	22 (22.68%)	19 (19.59%)	4 (4.12%)	0 (0%)	47	21	8е^-04^	
Annually	94 (52.22%)	12 (6.67%)	6 (3.33%)	3 (1.67%)	41 (22.78%)	17 (9.44%)	5 (2.78%)	2 (1.11%)				
Several years	21 (44.68%)	0 (0%)	12 (25.53%)	0 (0%)	9 (19.15%)	3 (6.38%)	2 (4.26%)	0 (0%)				
Emergency	48 (52.75%)	5 (5.5%)	6 (6.59%)	1 (1.1%)	19 (20.88%)	10 (10.99%)	1 (1.1%)	1 (1.1%)				
Overall	192 (46.27%)	26 (6.27%)	36 (8.68%)	6 (1.45%)	91 (21.93%)	49 (11.81%)	12 (2.89%)	3 (0.72%)				

## Discussion

The current survey was aimed at exploring the barriers to accessing dental services as perceived by the Bulgarian population and whether an association between these barriers and various demographic, socioeconomic, and psychosocial factors existed. The main results suggested that the leading barriers to accessing dental services in the Republic of Bulgaria originated from patients themselves. In our study, the barriers mainly occurred in women, people with higher levels of education and income, and rural populations. Barriers by state and society mainly occurred in men, those with low income, less educated individuals, 45-65 years old patients, and those visiting a dentist only in an emergency. Barriers by dental profession had the lowest impact on the access to services and were mainly registered among women, low-income people, and urban populations. These findings corresponded to previous results [9-12]. In addition, almost half of the participants reported the complex impact of more than one group of factors. In this aspect, multiple barriers were mainly suggested by middle-aged people (30-44 years), urban populations, patients with comorbidities, those rarely visiting a dental office, and those with a low self-assessment of dental status.

The leading reason for postponement of dental treatment according to almost a third of the study participants was lack of pain. This outcome corresponds to the results of a previous study in the Republic of Bulgaria in 2014 [12]. We also found that half of the patients delayed visits due to a lack of symptoms and negligence in seeking dental prevention. Lack of pain as a barrier was mainly registered among women, age group 45-65 years, and rural populations, which was in line with previous results [13-15].

Moreover, the cost of dental treatment was no longer found to be a significant factor in avoiding services in the Republic of Bulgaria. Furthermore, according to the present findings it had been overtaken by psychosocial factors such as lack of time and dental fear and anxiety. This was contrary to the results of other authors who reported the cost as the main barrier for patients [16-19]. The cost was mainly a limiting factor for women, middle-aged people, low-income individuals, and urban populations, which also corresponded to previous results [20-23].

Dental fear and anxiety were mainly reported by women, younger and less educated patients, those with low income, those with no income, people with low self-assessment of their oral health status, and those visiting dental offices irregularly. These findings confirmed the conclusions drawn by Folayan et al. [24] and Settineri et al. [25].

Negative experience with dental treatment was mainly registered by women and young and middle-aged persons. As suggested several times in the existing literature [11, 26-30], we also identified the main reasons for bad dental experience - failure in dental treatment and negligent attitude and behavior of the dental practitioner.

The complex impact of multiple barriers related to patients was found leading in all groups considering age, gender, education, income, general health status, self-assessment of oral health, frequency of visits, and being part of an urban population. As previously stated, lack of complaints was identified as a leading barrier only in rural populations in which there was no combined impact of multiple factors. Although our results focused on comparisons between complex and single factor influence, the authors did not find any research suggesting similar data.

This study provides valuable results on the leading barriers to accessing dental services and their associated factors; however, it has some limitations. First, when using sociological methods (e.g., questionnaires, as in the present survey), there is always a doubt if the respondents have answered honestly. In light of these considerations, the current outcomes should be interpreted with caution. Second, the cross-sectional design of the study provides only a snapshot of the current situation. Therefore, the longitudinal design of future research could better capture dynamics over time and reveal trends in the development of explored phenomena. Third, the small sample size might have affected the comprehensiveness of the results and may hinder the generalization of the data. Accordingly, future research should attempt to increase the number of participants, and it may be worth considering expanding the sample by including individuals under 18 and over 65 years. Finally, this study reported only national data, and in this aspect, further investigations may be conducted in other countries that could allow making comparisons on a multinational level.

Despite these limitations, this study suggests a useful research model for future studies to explore barriers to accessing dental services perceived by patients. Understanding the way that obstructing (limiting) factors impact patients’ health and well-being could improve utilization and quality of dental services.

## Conclusions

The leading group of barriers to accessing dental services in the Republic of Bulgaria were those that were patient-related. They were reported mainly by women, people with higher education and income, and those from a rural population. Barriers from state and society were indicated mainly by men, low-income people, the less educated, and elderly people. Barriers from dental profession had the lowest impact on the access to services. Complex impact by more than one group of factors was registered mainly among middle-aged persons, those from urban populations, those who rarely visited a dental office, and those with low self-assessment of their dental status.

Patients postponed dental treatment mainly due to lack of pain, which was most significant among women, the elderly, and those from rural populations. The cost of dental treatment was no longer a significant factor, having been overtaken by psychosocial factors such as lack of time and dental fear and anxiety. Financial and psychosocial factors were most often barriers for women and low-income patients.
